# Soluble cMet levels in urine are a significant prognostic biomarker for diabetic nephropathy

**DOI:** 10.1038/s41598-018-31121-1

**Published:** 2018-08-24

**Authors:** Yong Chul Kim, Jung Nam An, Jin Hyuk Kim, Young-Wook Choi, Sohee Oh, Sang Ho Kwon, Mi-Young Lee, Junghun Lee, Jae-Gyun Jeong, Chun Soo Lim, Yon Su Kim, Seung Hee Yang, Jung Pyo Lee

**Affiliations:** 1 Department of Internal Medicine, Seoul National University Hospital, Seoul, Korea; 2 Department of Internal Medicine, Seoul National University Boramae Medical Center, Seoul, Korea; 3 Department of Biostatistics, Seoul Metropolitan Government-Seoul National University Boramae Medical Center, Seoul, Korea; 4 ViroMed Co., Ltd, Seoul, Korea; 5 Department of Internal Medicine, Seoul National University College of Medicine, Seoul, Korea; 6 Department of Medical Science, Seoul National University College of Medicine, Seoul, Korea; 7 Kidney Research Institute, Seoul National University College of Medicine, Seoul, Korea; 8 Seoul National University Biomedical Research Institute, Seoul, Korea

## Abstract

Hepatocyte growth factor and its receptor cMet activate biological pathways necessary for repair and regeneration following kidney injury. Here, we evaluated the clinical role of urinary cMet as a prognostic biomarker in diabetic nephropathy (DN). A total of 218 patients with DN were enrolled in this study. We examined the association of urine cMet levels and long-term outcomes in patients with DN. The levels of urinary cMet were higher in patients with decreased renal function than in patients with relatively preserved renal function (5.25 ± 9.62 ng/ml versus 1.86 ± 4.77 ng/ml, P = 0.001). A fully adjusted model revealed that a urinary cMet cutoff of 2.9 ng/mL was associated with a hazard ratio for end-stage renal disease of 2.33 (95% confidence interval 1.19–4.57, P = 0.014). The addition of urinary cMet to serum creatinine and proteinuria provided the highest net reclassification improvement. We found that in primary cultured human glomerular endothelial cells, TGFβ treatment induced fibrosis, and the protein expression levels of collagen I, collagen IV, fibronectin, and αSMA were decreased after administration of an agonistic cMet antibody. In conclusion, elevated levels of urinary cMet at the time of initial diagnosis could predict renal outcomes in patients with DN.

## Introduction

The presence and severity of chronic kidney disease (CKD) are the strongest predictors of adverse outcome in individuals with diabetes^1^. However, an adverse prognosis is not inevitable in patients with overt nephropathy. Some patients do not develop end-stage renal disease (ESRD) or die. Developing new ways to identify patients with a good prognosis from those with poor prognostic outcomes remains important for the management of individuals with diabetic nephropathy (DN).

Several recent articles have highlighted the potential importance of some molecules as biomarkers for progressive kidney disease^2–7^. CKD nevertheless progresses in a notable percentage of patients with type 2 diabetes. Thus, an accurate test that complements current medical approaches in predicting CKD progression is necessary.

Hepatocyte growth factor (HGF) is a key cytokine that plays a critical role in the regulation of cell proliferation, wound healing and tissue fibrosis^8,9^. The antifibrotic effect of HGF has been studied extensively in many animal models of CKD^10–12^.

cMet, the tyrosine kinase receptor for HGF, has also been shown to be critical for cell survival via stimulation of an array of downstream signaling pathways, resulting in cell proliferation, spreading, migration, and inhibition of apoptosis^8,13^. These pleiotropic events have generated profound interest regarding the potential therapeutic role of the HGF/Met pathway in animal models of kidney injury. Recent *in vivo* evidence also supports a role for the HGF/Met pathway as an antifibrogenic factor because injection of recombinant HGF protein or the HGF gene led to reduced renal myofibroblast activation and diminished tubulointerstitial fibrosis^10,14,15^.

A recent study demonstrated that elevated serum HGF levels in the presence of protein-energy wasting increased all-cause mortality risk in ESRD patients^16^; moreover, HGF could predict all-cause mortality and cardiovascular mortality in the general population^17^. Several studies showed that urinary HGF is not only a prognostic biomarker in CKD^18,19^ but also useful in clinical risk prediction for recovery after acute kidney injury (AKI)^20,21^, especially in critically ill patients^22^ and kidney transplant recipients^23^.

To the best of our knowledge, there are currently no data regarding the prognostic role of urinary cMet in CKD, including CKD manifesting due to DN. In this study, we aimed to identify the association between soluble cMet levels in urine at the time of the initial DN diagnosis and clinical manifestations. We also evaluated the role of urinary soluble cMet in the progression of renal function and patient survival.

## Results

### Baseline characteristics and urinary soluble cMet in CKD patients

The baseline characteristics of the study participants are shown in Table 1. The median age was 61.3 years old, and 138 patients were male. Serum creatinine was 3.09 ± 2.40 mg/dl, and estimated glomerular filtration rate (GFR) was 33.9 ± 28.1 ml/min/1.73 m^2^ as calculated by the CKD-EPI creatinine equation. The random urine protein creatinine ratio was 3.8 ± 4.6 g/g.

**Table 1:** Baseline characteristics of CKD patients.

	Total	1	2	3	4	5	P
Number of patients (n)	218	15	19	58	63	63	
Age (years)	61.3 ± 13.9	41.6 ± 17.9	55.2 ± 16.0	64.0 ± 10.3	66.4 ± 11.6	60.5 ± 12.8	<0.001
Sex, male (n [%])	138 (63.3)	10 (66.7)	11 (57.9)	41 (70.7)	36 (57.1)	40 (63.5)	
BMI (kg/m^2^)	24.0 ± 4.3	25.4 ± 4.8	25.0 ± 3.0	23.9 ± 3.9	24.8 ± 4.4	23.1 ± 4.7	
SBP (mmHg)	140.5 ± 28.4	133.9 ± 20.0	134.9 ± 22.2	143.2 ± 27.4	139.2 ± 27.9	142.2 ± 32.0	
DBP (mmHg)	75.1 ± 16.3	82.6 ± 14.4	79.0 ± 12.1	78.3 ± 14.9	73.1 ± 16.2	72.6 ± 18.0	
cMet (ng/ml)	3.8 ± 8.1	1.4 ± 1.6	1.1 ± 2.0	2.2 ± 5.9	2.2 ± 5.4	8.3 ± 11.8	<0.001
cMet/Creatinine (ng/mg)	7.7 ± 21.1	1.3 ± 1.5	1.7 ± 3.7	4.6 ± 16.0	4.1 ± 12.6	17.7 ± 31.9	<0.001
Blood hemoglobin (d/gl)	10.9 ± 2.2	12.9 ± 2.8	12.7 ± 2.9	11.8 ± 2.0	10.4 ± 1.4	9.7 ± 1.5	<0.001
White blood cell (/ul)	7.3 ± 1.9	7.7 ± 1.8	7.3 ± 1.6	7.3 ± 1.4	7.4 ± 2.4	7.0 ± 2.0	
**Serum**							
Calcium (mg/dl)	8.5 ± 0.8	8.9 ± 0.9	8.8 ± 0.6	8.7 ± 0.6	8.6 ± 0.6	8.0 ± 0.8	<0.001
Phosphorus (mg/dl)	4.0 ± 0.8	3.7 ± 0.7	3.9 ± 0.5	3.7 ± 0.6	3.8 ± 0.6	4.6 ± 1.0	<0.001
uric acid (mg/dl)	7.3 ±1.9	6.4 ± 2.0	5.5 ± 2.1	7.4 ± 2.0	7.7 ± 1.6	7.6 ± 1.5	0.004
Cholesterol (mg/dl)	176 ± 55	178 ± 53	209 ± 103	173 ± 51	173 ± 45	170 ± 47	
Albumin (g/dl)	3.7 ± 0.6	4.0 ± 0.8	3.7 ± 0.6	3.7 ± 0.6	3.8 ± 0.6	3.5 ± 0.5	0.023
urea (mg/dl)	35.9 ± 15.4	15.0 ± 3.2	22.6 ± 6.4	31.1 ± 14.4	36.6 ± 9.7	48.8 ± 13.8	<0.001
Creatinine (mg/dl)	3.09 ± 2.40	0.79 ± 0.11	1.01 ± 0.16	1.68 ± 0.30	2.64 ± 0.53	6.01 ± 2.55	<0.001
eGFR (ml/min/1.73m^2^)	33.9 ± 28.1	107.4 ± 13.7	75.6 ± 9.2	40.4 ± 7.5	22.2 ± 4.1	9.6 ± 3.0	<0.001
Urine PCR (g/g)	3.8 ± 4.6	2.0 ± 3.2	2.4 ± 4.1	3.2 ± 5.0	2.8 ± 3.5	6.1 ± 4.7	<0.001

Categorical variables were presented as n (%), and continuous variables were shown as mean ± standard deviations. BMI, body mass index; SBP, systolic blood pressure; DBP, diastolic blood pressure; eGFR, estimated glomerular filtration rate (by CKD-EPI creatinine equation); PCR, protein-to-creatinine ratio.

**Figure 1:**
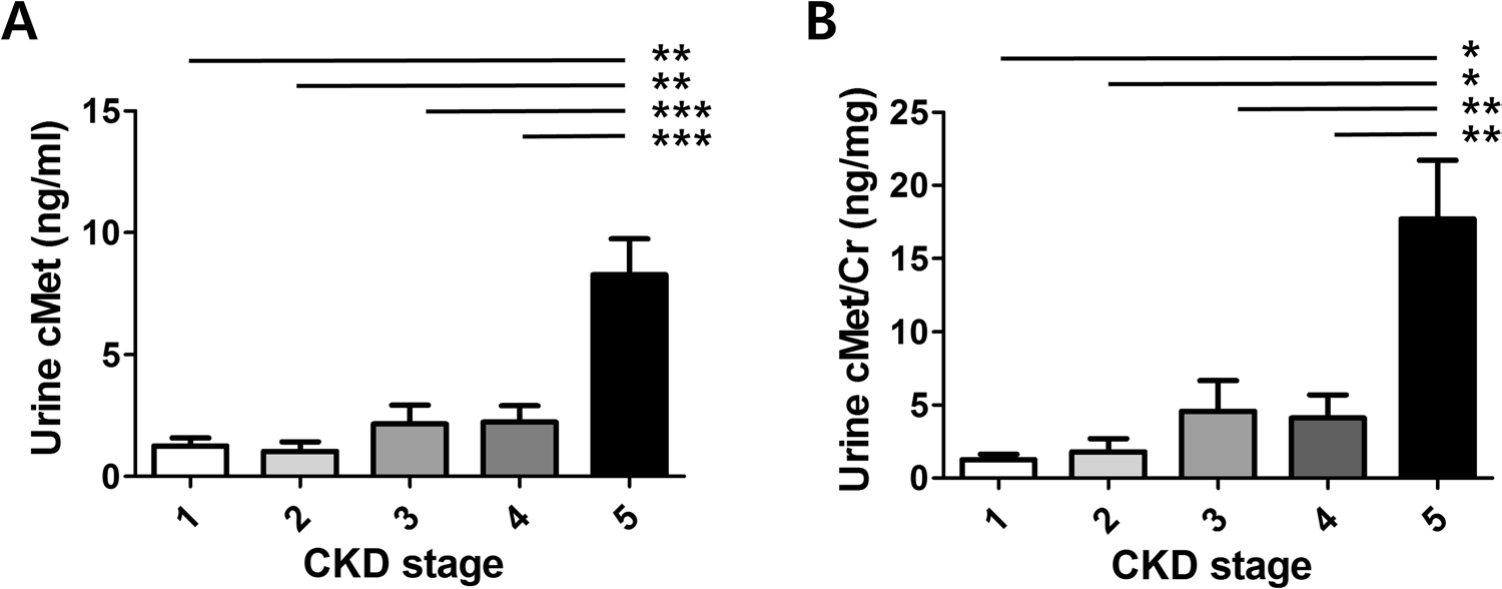
Correlation of urinary cMet levels with renal functions. *P < 0.05, **P < 0.01, ***P < 0.001.

Urinary soluble cMet levels as measured by enzyme-linked immunosorbent assay (ELISA) were significantly higher in patients with CKD stage V than in patients with CKD I, II, III, or IV (P < 0.001); this trend remained unchanged after urinary soluble cMet levels were adjusted for urinary creatinine (Fig. 1).

### Validation of urine cMet ELISA

To measure the concentration of soluble cMet protein present in a human urine sample with high accuracy and precision, a commercially available soluble cMet ELISA kit was used. To confirm the validity of the kit for our human urine study, several parameters such as the lower limit of quantification (LLOQ), dilution linearity, and parallelism were analyzed based on the FDA’s draft guidance document Bioanalytical Method Validation^24^, especially the standards regarding the ligand binding assay. To determine the LLOQ, two different batches of the ELISA kit (Lot nos: 1696848A2 and 1877133A) were purchased and employed. A series of two-fold dilutions were made using the calibrator provided in the kit starting from the highest concentration on the calibration curve (50 ng/ml). A total of 11 concentrations were prepared, and each concentration ranging from 0~50 ng/ml was run six times in a single assay to verify its accuracy and establish a coefficient of variation (CV) for precision. As shown in Supplementary Tables S1–3, 0.78 ng/ml was the lowest concentration in both batches that met acceptable accuracy and precision parameters within 25% of the nominal concentration and 25% CV. To further validate this value as the LLOQ, intra-assay (within an assay) and inter-assay (between assays) assessments were performed by two operators. The results showed that both parameters fell within the acceptable range of accuracy and CV, suggesting that 0.78 ng/ml can serve as the LLOQ.

To examine the matrix effect of human urine samples obtained from the patients with kidney disease, dilution linearity and parallelism were investigated at the laboratory scale (see Supplementary Table S4, Supplementary Fig. S1). For dilution linearity, six random urine samples were selected, each of which was subjected to threefold serial dilutions from 1 to 1/27 in standard diluent buffer and assessed for the levels of soluble cMet. Linear regression analysis relative to the expected concentration demonstrated strong dilution linearity in all samples with a correlation coefficient of 0.99 and a high level of accuracy (84~127%). To determine parallelism, the optical density of two samples with high endogenous levels of soluble cMet and their diluted concentrations was plotted against the soluble cMet calibration curve. The graph exhibited a robust parallel relationship between the natural soluble cMet and the cMet calibration curve. Taken together, these results suggest that there is no significant matrix effect observed in human urine samples.

### Association of clinical parameters with urine soluble cMet levels

**Figure 2:**
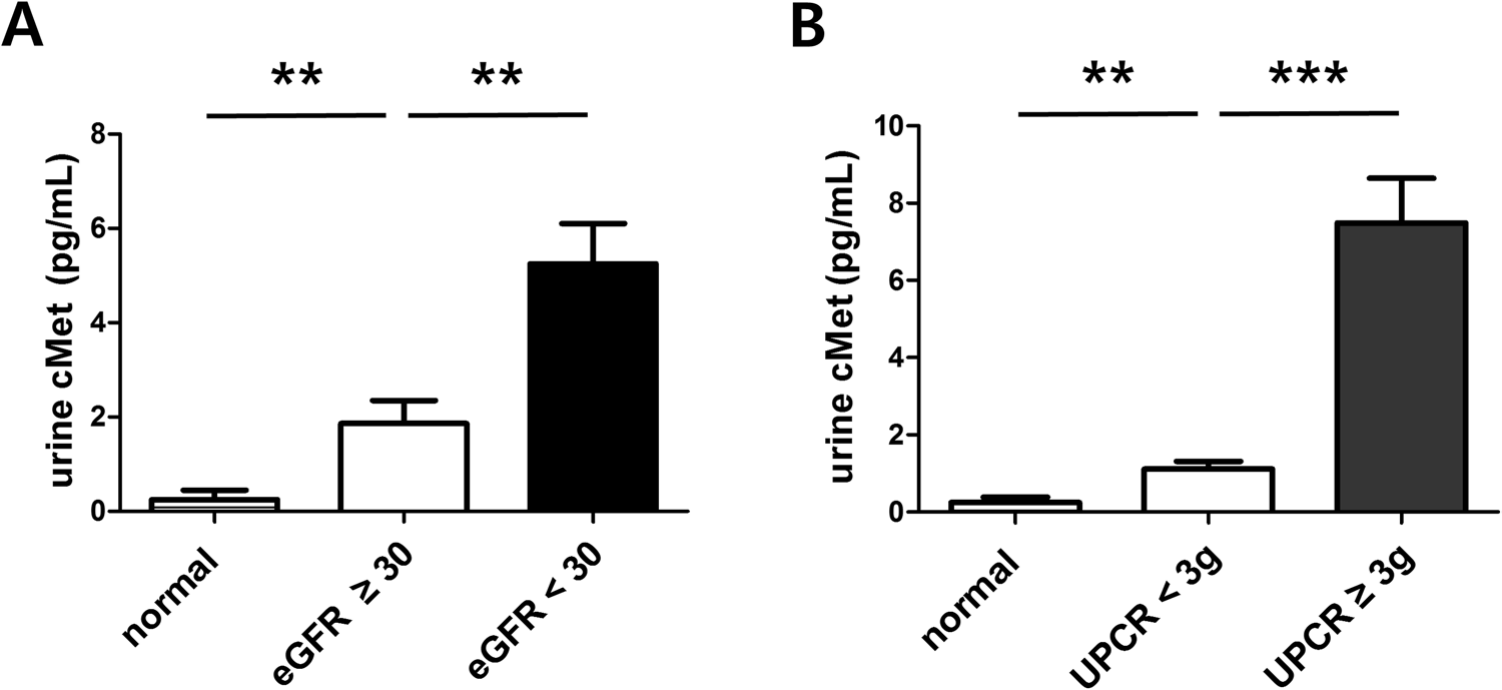
Urinary cMet levels stratified by renal function (**A**) and urine proteinuria (**B**). **P < 0.01, ***P < 0.001.

The levels of urine cMet were higher in patients with decreased renal function (<30 ml/min/1.73 m^2^) compared with patients with relatively preserved renal function (≥30 ml/min/1.73 m^2^) (5.25 ± 9.62 ng/ml versus 1.86 ± 4.77 ng/ml, P = 0.001, Fig. 2A). Moreover, urinary levels of cMet were also higher in patients with nephrotic proteinuria (≥3 g/g) than in those with sub-nephrotic proteinuria (7.58 ± 11.28 ng/ml versus 1.15 ± 2.28 ng/ml, P < 0.001, Fig. 2B).

### Association between urinary soluble cMet and clinical outcomes

**Figure 3:**
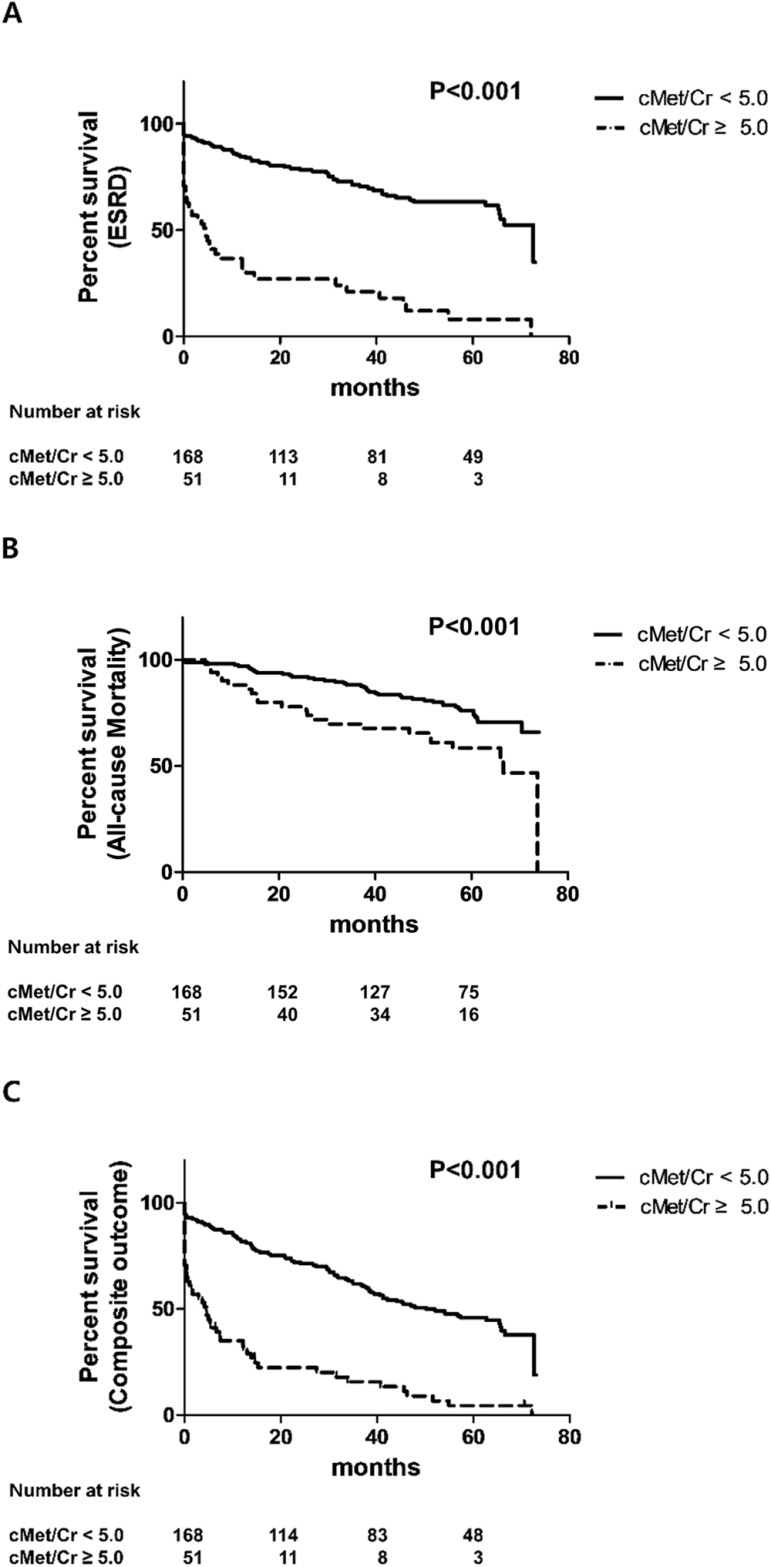
Kaplan-Meier patient survival curves for ESRD (**A**), all-cause mortality (**B**), and the composite outcome (**C**).

During the median follow-up of 49.8 ± 19.6 months, 101 (46.1%) patients were diagnosed with ESRD, and 65 (29.7%) patients died. To investigate the association between the urinary soluble cMet concentration and ESRD or mortality, we performed a Kaplan-Meier survival analysis. The risk of ESRD (Fig. 3A) and all-cause mortality (Fig. 3B) as well as the composite outcome (Fig. 3C) during the follow-up was increased in DN patients with higher urine cMet/Cr levels compared with those in DN patients with lower levels of urinary cMet (P < 0.001).

The independent effect of soluble urinary cMet on renal and patient outcomes was examined by using multivariate Cox proportional hazard models (Table 2). Elevated cMet values remained an independent variable associated with ESRD or the composite outcome after adjusting for confounding variables, including age, sex, hemoglobin, albumin, AST, ALT, uric acid, cholesterol, calcium, phosphorus, estimated glomerular filtration rate (eGFR), and the urine protein-to-creatinine ratio (UPCR) (ESRD: hazard ratio (HR) 2.33, 95% confidence interval (CI) 1.19–4.57, P = 0.014; Composite outcome: HR 2.14, 95% CI 1.18–3.86, P = 0.012; Death: HR 1.88, 95% CI 0.81–4.39, P = 0.142).

**Table 2:** Association of urine soluble cMet levels with graft failure and mortality using conventional Cox proportional hazards models.

	Unadjusted		Model 1		Model 2	
	HR (95% CI)	P	HR (95% CI)	P	HR (95% CI)	P
**ESRD**						
**cMet**						
cut-off value^a^	4.39 (2.92–6.59)	<0.001	3.75 (2.17–6.48)	<0.001	2.33 (1.19–4.57)	0.014
**cMet/Cr**						
cut-off value^a^	5.04 (3.35–7.59)	<0.001	4.73 (2.67–8.36)	<0.001	3.07 (1.66–5.69)	0.001
**Composite**						
**cMet**						
cut-off value^a^	3.71 (2.59–5.32)	<0.001	3.17 (1.94–5.19)	<0.001	2.14 (1.18–3.86)	0.012
**cMet/Cr**						
cut-off value^a^	4.16 (2.89–6.00)	<0.001	3.41 (2.02–5.76)	<0.001	2.11 (1.20–3.71)	0.009
**Mortality**						
**cMet**						
cut-off value^a^	2.13 (1.29–3.52)	0.003	2.42 (1.00–5.84)	0.05	1.88 (0.81–4.39)	0.142
**cMet/Cr**						
cut-off value^a^	2.07 (1.24–3.45)	0.005	2.42 (1.09–5.38)	0.031	1.96 (0.86–4.46)	0.11

Multivariate Cox proportional hazard ratios.

Model 1: adjusted for age, sex, hypertension, Hb, albumin, AST, ALT, uric acid, cholesterol, Ca, P,

Model 2: adjusted for age, sex, hypertension, Hb, albumin, AST, ALT, uric acid, cholesterol, Ca, P, GFR, PCR

HR, hazard ratio; CI, confidence interval.

^a^Analyses were computed by including the predefined cut-off value of urine soluble cMet and cMet/Cr according to the ROC curve; both cut-off values were 2.9 ng/ml and 4.9 ng/mg with regard to mortality and end stage renal disease (ESRD).

### Added value of urine soluble cMet/Cr in predicting adverse outcomes

Next, we determined whether urine soluble cMet/Cr is a better indicator of ESRD than serum Cr and UPCR by comparing the receiving operating characteristic (ROC) curves (Delong test), integrated discrimination improvement (IDI) index and the category-free net reclassification improvement (cfNRI) index. The AUCs (95% CI) of Cr (model 1), Cr + UPCR (model 2) and Cr + UPCR + cMet (model 3) were 0.858 (0.808–0.907), 0.813 (0.755–0.870), and 0.890 (0.848, 0.933), respectively. The addition of urine soluble cMet/Cr to serum Cr or serum Cr + UPCR statistically improved the predictive value for ESRD and the composite outcome but not death (Table 3). The IDI and cfNRI for predicting ESRD (model 1 vs model 3) were 6.76% (95% CI 3.38–10.15, P = 0.0001) and 82.64% (95% CI 59.07–106.22, P < 0.0001), respectively, suggesting that measuring urinary soluble cMet substantially increases the predictive value for adverse outcomes.

**Table 3:** Comparison of the ROC curve, IDI and category-free NRI of the Cr vs. Cr, UPCR *vs*. Cr, UPCR, cMet/Cr in predicting ESRD.

	AUC (95% CI)	DeLong test	IDI		category-free NRI	
		P-value	P-value	(95% CI)	P-value	(95% CI)
**ESRD**						
Cr	0.858 (0.808, 0.907)	Reference	Reference		Reference	
cMet/Cr	0.694 (0.623, 0.765)	<0.0001	<0.0001		<0.0001	
Cr+UPCR	0.889 (0.846, 0.931)	0.0389	0.0001	6.52% (3.18%, 9.87%)	<0.0001	88.31% (65.02%, 111.61%)
Cr+UPCR+cMet/Cr	0.890 (0.848, 0.933)	0.0295	0.0001	6.76% (3.38%, 10.15%)	<0.0001	82.64% (59.07%, 106.22%)
**Death**						
Cr	0.644 (0.569, 0.720)	Reference	Reference		Reference	
cMet/Cr	0.620 (0.536, 0.704)	0.5943	0.0748		0.1726	
Cr+UPCR	0.633 (0.556, 0.710)	0.5717	0.4641	0.32% (−0.53%, 1.17%)	0.3923	11.95% (−15.42%, 39.31%)
Cr+UPCR+cMet/Cr	0.641 (0.558, 0.724)	0.9138	0.0372	2.76% (0.16%, 5.35%)	0.8453	−2.82% (−31.11%, 25.47%)
**Composite**						
Cr	0.865 (0.818, 0.911)	Reference	Reference		Reference	
cMet/Cr	0.694 (0.626, 0.761)	<0.0001	<0.0001		<0.0001	
Cr+UPCR	0.878 (0.835, 0.922)	0.149	0.0196	2.86% (0.46%, 5.27%)	<0.0001	81.06% (57.53%, 104.59%)
Cr+UPCR+cMet/Cr	0.886 (0.844, 0.928)	0.0555	0.0005	4.78% (2.10%, 7.46%)	<0.0001	74.21% (50.30%, 98.12%)

Cr, creatinine; UPCR, urine protein-to-creatinine ratio; ESRD, end stage renal disease; CI, confidence interval; IDI, integrated discriminatory improvement; NRI, net reclassification improvement.

### Recombinant HGF and agonistic cMet antibody therapy modulates fibrosis in glomerular endothelial cells

**Figure 4:**
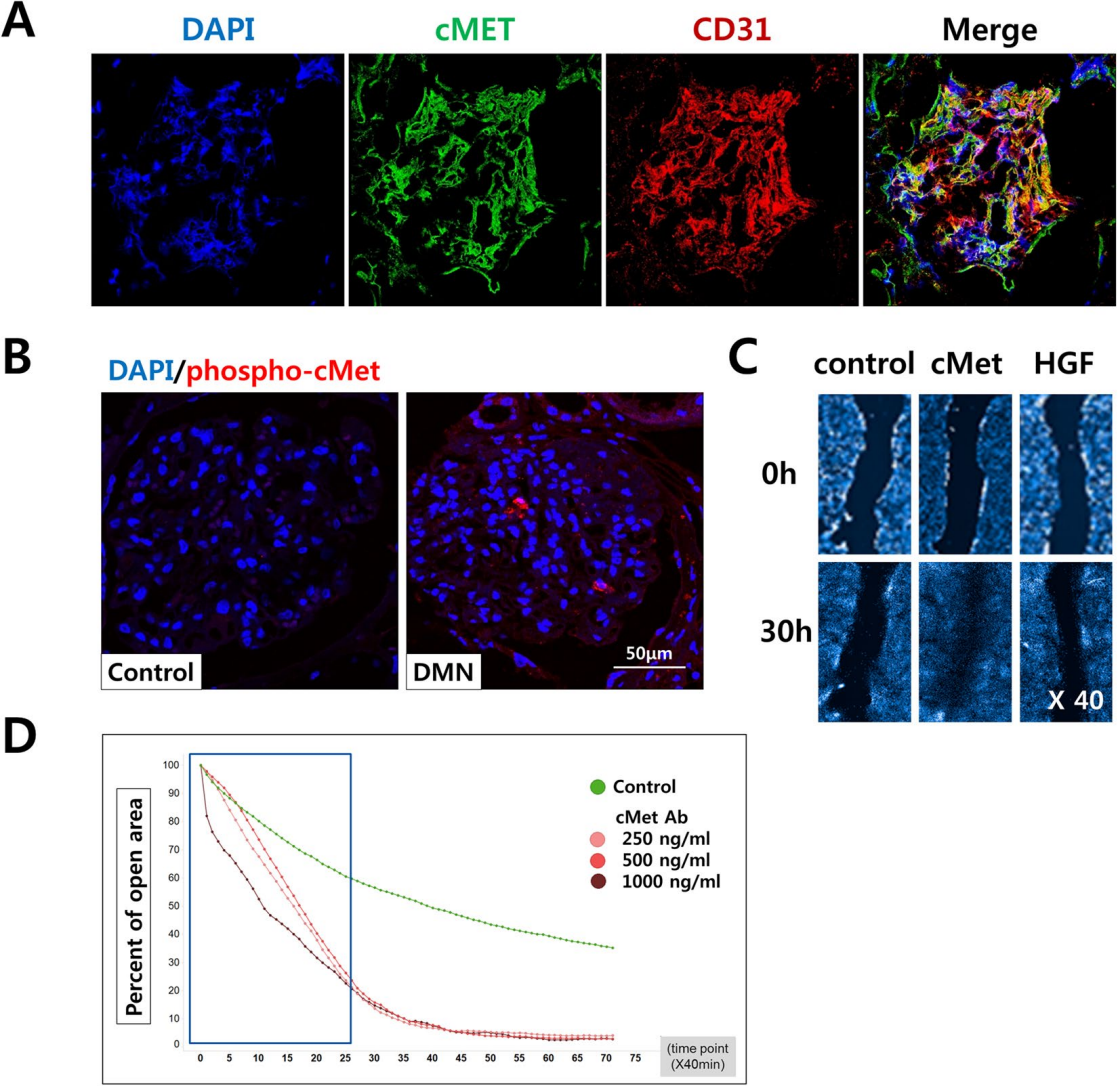
Glomerular endothelial cells *in vitro* and wound healing analysis. (**A**) Immunofluorescence for DAPI (blue), cMet (green), and CD31 (red) was observed in healthy human glomeruli. Original magnification: X400. (**B**) The levels of phosphorylated cMet were increased in the glomeruli from patients with diabetic nephropathy compared with those from normal control subjects; DAPI (blue), phospho-cMet (red). Original magnification: X400. (**C**) Assessment of the endothelial cell migratory capacity was assessed using a scratch wound healing assay between 0 and 30 hours. There was a significant difference in the migratory potential of the cMet group compared to the control and HGF groups.

We found that glomerular endothelial cells (GECs) expressing cMet were abundant in the healthy glomerulus (Fig. 4A). Next, we investigated whether the HGF/Met pathway is involved in renal fibrosis in DN patients using immunofluorescence staining with a phosphorylation-specific cMet antibody (Ab) to determine the levels of active cMet. As shown in Fig. 4B, cMet was activated in patients with DN. The wound healing assay is a well-established procedure to study cell migration *in vitro*. GECs were plated in 48-well plates after a 2nd passage and cultured to establish a monolayer. After a 6-hour serum starvation step, the monolayer was scratched with a p20 pipette tip, and movement of cells into the scratched area was monitored using immunofluorescence and bright-field microscopy at 0 and 30 hours after scratching (Fig. 4C). During the 30-hour period, the cells treated with cMet Ab progressed into the wound area more rapidly than untreated cells (Fig. 4D).

**Figure 5:**
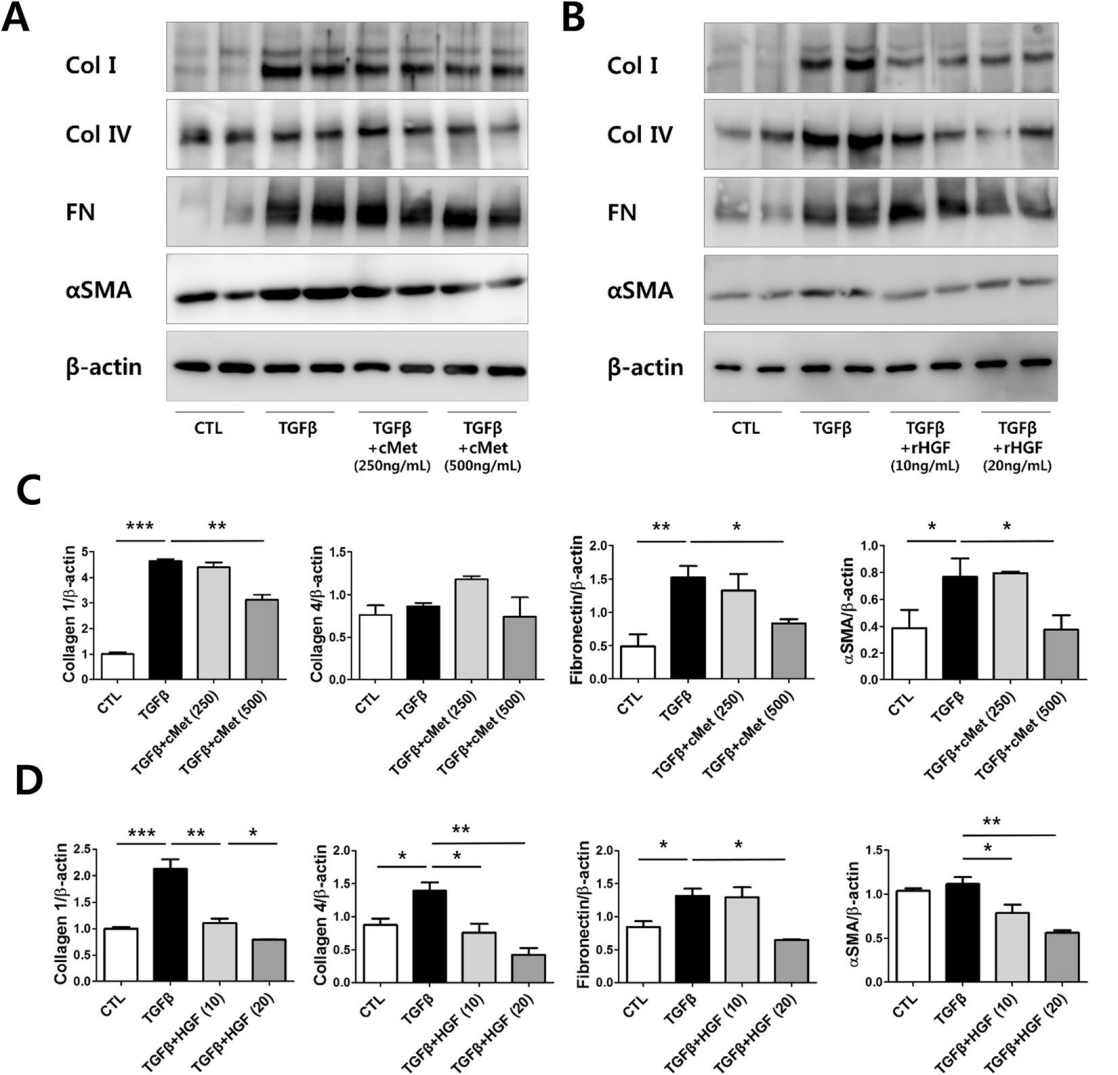
Agonistic cMet Ab and rHGF protects human glomerular endothelial cells (GECs) against fibrosis in an *in vitro* model. (**A**–**D**) After fibrosis induction with TGF-β in GECs, agonistic cMet Ab (**A**,**C**) and rHGF (**B**,**D**) ameliorated the fibrosis. The cropped blots are used in the figure, and full-length blots are presented in Supplementary Figs S2–S3, respectively. All values are presented as the means ± SEM (*n* = 4 per group for each experiment). *P < 0.05, **P < 0.01.

We hypothesized that the HGF/Met pathway could aggravate in fibrosis induced kidney cells *in vitro* experiments. To assess the role of the HGF/Met pathway in kidney cell fibrosis, we treated primary cultured human GECs with TGFβ to induce fibrosis. Remarkably, the protein expression levels of collagen I, collagen IV, fibronectin and αSMA were upregulated after TGFβ treatment indicating that fibrosis was adequately induced. GECs treated with TGFβ and either cMet Ab (Fig. 5A,C) or rHGF (Fig. 5B,D) displayed significantly downregulated protein expression of collagen I, collagen IV, fibronectin and αSMA.

## Discussion

To the best of our knowledge, this is the first study to demonstrate that elevated urinary of soluble cMet levels in patients with diabetic nephropathy (DN) are correlated with renal function. More importantly, we report that increased urine soluble cMet levels are significantly associated with renal survival and mortality. Inclusion of urinary soluble cMet in the fully adjusted ESRD models improved their predictive power with three different statistical methods (C-statistics, IDI and NRI). Therefore, urine soluble cMet levels at diagnosis may serve as a novel biomarker to predict the renal progression in DN.

More than 380 million people currently suffer from diabetes. The International Diabetes Federation estimated that this would rise to 592 million people within a generation. Despite the continuous increase in the prevalence of type 2 diabetes worldwide, there has been a gradual decline in mortality from macrovascular complications such as stroke and coronary heart disease owing to the advent of potent statins. However, this is not the case in DN because end-stage renal disease (ESRD) remains a global leading cause in individuals with DN, and its incidence is still rising. Notably, once nephropathy develops, approximately 20–40% of patients inevitably progress to ESRD. Therefore, we need biomarkers that would enable early risk stratification in DN^25^.

In the past, persistent microalbuminuria was the most studied biomarker in DN. Both the presence and incremental changes in microalbuminuria have been shown to correlate with the development and progression of chronic kidney disease (CKD) in type 1 and type 2 diabetes^26^. Of the emerging candidate biomarkers, serum cystatin C^4^, fibroblast growth factor 23 (FGF23)^7^ and soluble tumor necrosis factor (TNF) receptors^2^ have provided the most promising data.

The HGF/Met pathway has been shown to play a critical role in glomerular and tubulointerstitial fibrosis in a diabetic mouse model^9^. Recombinant HGF treatment reversed fibrotic changes induced by streptozocin injection. rHGF also repressed TGFβ production, which plays a key role in renal fibrosis in glomerular mesangial cells even under hyperglycemic conditions. Moreover, intravenous administration of HGF not only attenuated urine protein excretion but also mitigated mesangial expansion, reduced collagen deposition, and prevented interstitial myofibroblast activation in type I^27^ and type II^28^ DN mouse models. A recent study demonstrated that HGF enhances β-cell proliferation and ameliorates hyperglycemia by maintaining compensatory hyperinsulinemia^29^. In the present study, administration of either agonistic cMet Ab or rHGF in primary cultured GECs downregulated the expression of fibronectin, collagen I, collagen IV and αSMA, which was consistent with the results of previous studies. Recently, the endothelial-to-mesenchymal transition (EndMT), in which endothelial cells acquire mesenchymal characteristics, has also been recognized as a crucial mechanism of renal fibrosis^30,31^, especially in diabetic nephropathy mouse models^32,33^. Therefore, regulation of endothelial dysfunction in the kidney might contribute to attenuate renal fibrosis and further experiments with DN mouse models to explore the efficacy of cMet agonistic Ab is needed in the future.

Several studies have reported that serum HGF is associated with increased mortality in patients with CKD^16^, but the role of soluble cMet levels in predicting renal failure has not been studied in detail. In addition, there are no data regarding urinary soluble cMet levels. Previous studies have shown that soluble form of cMet could be generated via shedding and cleavage by γ-secretase: Sheddases or metalloproteinases cleave full-length cMet within its extracellular domain, resulting in different in a soluble extracellular N-terminal fragment (Met-NTF). We have demonstrated that cMet expression is increased in GECs, therefore it is reasonable to infer that soluble cMet in the urine is originated from the kidney^34,35^. Urine is a useful source of protein for biomarker analysis because it can be obtained by non-invasive methods. Therefore, using urine samples to identify novel biomarkers may have clinical implications. However, urine specimens demonstrate a high degree of variability in volume, protein concentration, total protein excreted, and pH and can degrade upon storage^36,37^. To account for these potential issues, we validated the cMet ELISA kit with several parameters such as the LLOQ, dilution linearity, and parallelism.

In our study, urinary cMet/Cr levels at the time of DN diagnosis were significantly correlated with UPCR and negatively correlated with eGFR. However, cMet/Cr was predominantly increased in CKD stage V patients, but there was no difference in the other CKD stages. These results suggest that cMet filtration is simply increased as kidney function deteriorates. To confirm this hypothesis, we adjusted the cMet levels with the urinary creatinine levels.

Compared with conventional prognostic markers such as serum creatinine and UPCR, cMet alone showed moderate utility as a biomarker in the ROC analysis. To improve the power of ESRD prediction, we evaluated the additive predictive performance of urine cMet/Cr at the time of DN diagnosis. Patients who developed ESRD had relatively high levels of urinary soluble cMet/Cr.

Modern improvements in diabetes care and cardiovascular survival have allowed many patients with diabetes to live long enough to require renal replacement. One key strength of this study is the large number of ESRD events in our cohort compared with previous studies of patients with DN^2,38^. In our cohort, 45% of participants developed ESRD during follow-up (n = 99 patients out of 218). This might be due to the relatively longer duration of the follow-up period (31.0 ± 22.4 months). Another strength is that this is the first article to show the prognostic effect of urinary soluble cMet, and we validated the ELISA kit for the LLOQ and the matrix effect of human urine samples.

However, the present study has some limitations. First, biopsy samples of DN were insufficient. Only some of the patients had biopsy-proven DN, so the time point of for the initial DN diagnosis might be not consistent. Since the diagnosis of DN is made by clinical judgment rather than histological examination, this problem is be a common limitation of DN studies. Nevertheless, this is the first article to prove the predictive effect of urinary soluble cMet in DN. Second, there were no data regarding the serum levels of soluble cMet. Identifying whether there exists any correlation between serum and urinary soluble cMet levels would be helpful, and studies addressing this are expected in the future.

In conclusion, elevated levels of urinary soluble cMet at the time of initial diagnosis could predict renal outcome in patients with DN. The results suggest a critical role of cMet in renal fibrosis in CKD patients, especially those with DN.

## Methods

### Study population and data sources

The present study was conducted with the approval of the Research Ethics Committee of the Seoul National University Boramae Medical Center. All procedures were performed in accordance with the ethical standards of the institutional and/or national research committee and with the 1964 Declaration of Helsinki and its later amendments or comparable ethical standards. Informed consent was obtained to human research participants for the use of blood, urine samples as well as tissue samples.

In this study, 218 patients with clinically diagnosed DN were enrolled between January 2009 and December 2016. Among 218 patients, 20 patients had biopsy-proven disease. Patients under 18 years or who were planning to undergo kidney transplantation were excluded. Demographic and clinical characteristics at the time of diagnosis, including age, gender, body mass index (BMI), systolic and diastolic blood pressure, and comorbidities (e.g., hypertension, diabetes mellitus) were extracted from electronic medical records.

### Laboratory measures

Laboratory values, including complete blood cell counts, hemoglobin and serum levels of AST, ALT, albumin, urea, creatinine, calcium, phosphorus, cholesterol, glucose, and HbA1c, were collected. Urinary protein and creatinine levels were also collected, and the urinary protein-to-creatinine ratio (UPCR) was calculated.

Laboratory values were determined using the following instrumentation: a Modular D2400 analyzer with ISE900 module (Hitachi Ltd., Tokyo, Japan) and a cobas 8000 modular analyzer (Roche Diagnostics, Basel, Switzerland). The eGFR was calculated by the CKD-EPI creatinine equation^39^.

### Measurement of urine soluble cMet

A soluble cMet ELISA (catalog no. KHO 2031) kit was obtained from Thermo Fisher Scientific Inc. (Waltham, MA, USA). The ELISA was performed according to the manufacturer’s instructions. The LLOQ for soluble cMet was 0.78 ng/ml.

### Clinical outcomes

The clinical outcomes assessed were ESRD, all-cause mortality and a composite of the two outcomes. ESRD was defined as an irreversible loss of renal function with the need to start dialysis or to undergo renal transplantation. The mortality data of patients who were lost to follow-up were obtained from the National Statistical Office database.

### Histologic examination

After kidneys were fixed with paraformaldehyde (4%) and paraffin embedded, they were sliced into 4-μm sections and stained. The sections were then deparaffinized in xylene and rehydrated in a graded series of ethanol. Endogenous peroxidase activity was blocked with 0.3% hydrogen peroxidase in methanol for 30 minutes at room temperature. Sections were microwaved for 30 minutes in an antigen unmasking solution for antigen retrieval. After the sections were incubated with 10% goat serum for 1 hour at room temperature, they were incubated at 4 °C with anti-Met (cMet) antibody (Abcam, Cambridge, UK) and anti-Met (cMet) (phospho Y1349) antibody (Abcam) overnight. Tissues were washed several times in phosphate-buffered saline (PBS) and incubated for 40 minutes with Alexa Fluor-conjugated secondary antibodies (Molecular Probes, Eugene, OR). 4′,6-Diamidino-2-phenylindole (DAPI; Molecular Probes) was used to counterstain the nuclei. The negative control sections underwent the same treatment regimen except for the omission of primary antibodies. Sections were visualized using confocal microscopy with a Leica TCS SP8 STED CW (Leica, Wetzlar Germany).

### Cell culture of primary GECs

We isolated GECs from normal adjacent kidney tissue from patients with renal cell carcinoma according to guidelines approved by The Institutional Review Board of Seoul National University Hospital (IRB no. 1404-117-515). Kidneys were obtained from freshly sacrificed mice perfused through the heart with cold Hank’s balanced salt solution (HBSS), and glomeruli were prepared by a serial sieving method. Minced renal cortex tissue was serially passed through 160, 120 and 100 μm mesh stainless steel screens, and glomeruli were collected using a 75 μm mesh screen. The glomeruli, which contain small debris from the renal tubule, were suspended in HBSS (Gibco-BRL Life Technologies, Gaithersburg, MD), washed twice by brief centrifugation (800 × g, 1 min) and digested in 1.5 mg/ml collagenase (Sigma, St Louis, MO) for 40 minutes at 37 °C with occasional vortexing. Undigested glomeruli were pelleted by brief centrifugation, and the supernatant containing a single-cell suspension of GECs was resuspended in growth medium [RPMI-1640 (Sigma) supplemented with 20% heat-inactivated fetal bovine serum (FBS, Gibco-BRL), 5 ng/ml vascular endothelial growth factor (VEGF) (Peprotech, Rocky Hill, NJ), 10 ng/ml bFGF (Sigma), 10 ng/ml epidermal growth factor (EGF) (Sigma), 20 U/ml heparin, 1 mg/ml hydrocortisone (Sigma), 50 U/ml penicillin and 50 mg/ml streptomycin (Gibco-BRL)] and plated on fibronectin-coated 35-mm dishes (Becton Dickinson, Franklin Lakes, NJ). Small colonies of GECs were observed within 1 week after plating. To remove contaminating mesangial and epithelial cells, brief trypsinization was performed until a culture with 98.1% endothelial cells was achieved. The cells were maintained in collagen-coated 100-mm culture dishes (Iwaki, Tokyo, Japan)^40^. After 3 days of culture, cells were detached from the dishes with a 3 mM EDTA solution and a minimal amount of trypsin. Cells (2 × 10^5^/well) were seeded on 8-well chamber slides with serum-free medium for 24 hours and then washed twice with PBS. Next, recombinant TGFβ (R&D Systems, Minneapolis, MN, USA) was added at a final concentration of 10 ng/ml except where otherwise indicated. Cells were incubated in the presence or absence of either recombinant HGF (10 and 20 mg/mL) or agonistic cMet antibody (250 and 500 ng/mL) for 48 hours.

### Wound healing assay

GECs from the 2nd or 3rd passage were seeded into a 96-well plate (5 × 10^4^ cells/well) in medium supplemented with 10% FBS and left to grow into a confluent monolayer. To assay the basal cell migration ability of GECs, the confluent cell monolayer was serum-starved for 12 hours, after which a wound was scratched on the monolayer using a p20 pipette tip. The cell-free area was measured immediately (0 hours) and 30 hours after the scratch. To study the GEC response to cMet Ab and rHGF treatment, we cultured cells in medium supplemented with 10% FBS for 48 hours and then serum-starved the cells for 6 hours. The cells were subsequently treated with DMSO (control), medium containing cMet Ab (500 ng/mL), or rHGF (50 ng/mL). To visually assess viability, DAPI (Molecular Probes) was added, and cells were observed with a high-content imaging device (Operetta CLS; Perkin-Elmer, Germany) at different time points following treatment.

### Western blot analysis

GECs were lysed in RIPA buffer [50 mM Tris·HCl, pH 7.3; 150 mM NaCl; 0.1 mM EDTA; 1% (vol/vol) sodium deoxycholate; 1% (vol/vol) Triton X-100; 0.2% NaF; and 100 µM Na_3_VO_4_] supplemented with cOmplete protease inhibitors (Roche Applied Science, Indianapolis, IN). The kidney homogenate was centrifuged at 12,000 *g* for 30 min at 4 °C, and the protein concentration of the supernatant was determined by the Bradford method. Different amounts of extracted protein were separated by SDS-PAGE and transferred onto Immobilon-FL 0.4-μm polyvinylidene difluoride membranes (Millipore). Tris-buffered saline containing 0.1% Tween 20 was used as the wash buffer. After the membranes were treated with primary antibodies, anti-rabbit (1:5,000; Cell Signaling Technology, Danvers, MA) and anti-mouse (1:6,000 for β-actin; Cell Signaling Technology) secondary antibodies were used as appropriate. Detection of labeled proteins was performed with an enhanced chemiluminescence system (ECLTM PRN 2106; Amersham Pharmacia Biotech, Buckinghamshire, UK). The band intensities were analyzed using a gel documentation system (Bio-Rad Gel Doc 1000 and Multi-Analyst version 1.1).

Immunoblotting was performed using primary antibodies against collagen-1 (Abcam), collagen-4 (Abcam), fibronectin (Santa Cruz Biotechnology, Dallas, TX), αSMA (Abcam), and β-actin (Cell Signaling Technology).

### Statistical analysis

The patients were classified into two groups using either a urinary of cMet cutoff of 2.9 ng/ml or a cMet/Cr cutoff of 4.9 ng/mg, which were calculated with ROC curves. Categorical variables, which were expressed as frequencies and proportions, were compared using chi-square tests. After testing for normality, normally distributed continuous variables were expressed as the mean ± standard deviations and were compared using either Student’s t-test or one-way ANOVA test. The non-normally distributed variables were expressed as medians (25th, 75th percentiles) and were compared using the Mann-Whitney U or Kruskal-Wallis test.

To investigate the impact of urinary cMet or cMet/Cr levels on ESRD and mortality, Kaplan-Meier survival curves as stratified by the levels of urinary cMet or cMet/Cr were compared with log-rank tests.

Cox proportional hazard models with time-fixed urinary cMet or cMet/Cr levels at the time of DN diagnosis were used for multivariate survival analyses. Significant covariates identified in the univariate analysis and clinically important covariates were included in the final multivariable-adjusted analysis, which was conducted in a backward stepwise manner. The adjusted covariates for each model were as follows: Model 1, age, sex, hypertension, Hb, albumin, AST, ALT, uric acid, cholesterol, Ca, and P; and Model 2, age, sex, hypertension, Hb, albumin, AST, ALT, uric acid, cholesterol, Ca, P, GFR, and PCR. HR, hazard ratio; CI, confidence interval.

To examine the incremental prognostic value before and after inclusion of urinary soluble cMet levels to the traditional variables such as serum creatinine and the UPCR, we calculated the statistical significance of the difference between the areas under the two ROC curves (AUCs) with the method described by DeLong *et al*.^41^. Additionally, changes in the predictive accuracy of the models were evaluated by calculating the relative IDI and the cfNRI as described by Pencina *et al*.^42^.

All statistical analyses were performed using SPSS version 22 (IBM, Chicago, Illinois, United States) and R (version 3.2.5; The R Foundation for Statistical Computing, Vienna, Austria). A *P* value less than 0.05 indicated statistical significance.

## Electronic supplementary material

Supplementary Information

## Data Availability

The datasets generated during and/or analyzed during the current study are available from the corresponding author on reasonable request.
