# Supplementary material for: Soluble cMet levels in urine are a significant prognostic biomarker for diabetic nephropathy
Source: Sci Rep. 2018 Aug 24;8:12738. doi: 10.1038/s41598-018-31121-1 (PMC6109090; doi:10.1038/s41598-018-31121-1)
Supplement: Supplementary file 1 — Supplementary Information [file 41598_2018_31121_MOESM1_ESM.pdf]

Soluble cMet levels in urine are a significant prognostic biomarker for diabetic nephropathy

Yong Chul Kim[1], Jung Nam An[2], Jin Hyuk Kim[2], Young-Wook Choi[2], Sohee Oh[3], Sang Ho Kwon[4], Mi-Young Lee[4], Junghun Lee[4], Jae-Gyun Jeong[4], Chun Soo Lim[2,5], Yon Su Kim[6,7], Seung Hee Yang[7,8*], Jung Pyo Lee[2,5*].

*Both authors contributed equally to this work.

Author Affiliations

[1]*Department of Internal Medicine, Seoul National University Hospital, Seoul, Korea.*

[2]*Department of Internal Medicine, Seoul National University Boramae Medical Center, Seoul, Korea.*

[3]*Department of Biostatistics, Seoul Metropolitan Government-Seoul National University Boramae Medical Center, Seoul, Korea*

[4]*ViroMed Co., Ltd., Seoul, Korea.*

[5]*Department of Internal Medicine, Seoul National University College of Medicine, Seoul, Korea.*

[6]*Department of Medical Science, Seoul National University College of Medicine, Seoul, Korea.*

[7]*Kidney Research Institute, Seoul National University College of Medicine, Seoul, Korea.*

[8]*Seoul National University Biomedical Research Institute, Seoul, Korea.*

Correspondence:

Seung Hee Yang, PhD

Associate Professor

Seoul National University Biomedical Research Institute and Kidney Research Institute, Seoul National University

101 Daehak-ro, Jongno-gu, Seoul, 03080, Republic of Korea

E-mail: ysh5794@gmail.com

and

Jung Pyo Lee, MD, PhD

Associate Professor

Department of Internal Medicine, Seoul National University Boramae Medical Center and Seoul National University College of Medicine

20 Boramae-ro 5-gil, Dongjak-gu, Seoul 07061, Republic of Korea.

E-mail: nephrolee@gmail.com

Supplementary Table S1. Establishment of Lower Limit of Quantification (LLOQ), Lot No.1696848A2

Concentration (ng/ml)	Mean O.D. at 450 nm (n=6)	Mean Back-calculated Concentration (ng/ml)	Standard Deviation	Accuracy (%)	Precision (CV%)
50	2.419	49.91	0.117	100	10
25	1.682	25.74	0.104	103	10
12.5	0.986	11.93	0.061	95	8
6.25	0.598	6.27	0.046	100	10
3.12	0.369	3.39	0.025	108	9
1.56	0.216	1.60	0.012	102	9
0.78	0.144	0.78	0.008	100	11
0.391	0.107	0.37	0.009	95	26
0.195	0.089	0.17	0.01	87	67
0.098	0.090	0.18	0.012	182	73
0	0.070	NA	NA	NA	NA

The lowest limit of quantification (LLOQ) of urine soluble cMet was experimentally determined to be 0.78 ng/ml along the calibration curve. This value showed the acceptable level of accuracy and precision in both batches of the ELISA kit.

Supplementary Table S2. Establishment of Lower Limit of Quantification (LLOQ), Lot No.1877133A

Concentration (ng/ml)	Mean O.D. at 450 nm (n=6)	Mean Back-Calculated Concentration (ng/ml)	Standard Deviation	Accuracy (%)	Precision (CV%)
50	2.905	49.88	0.046	100	3
25	1.971	25.32	0.064	101	5
12.5	1.176	12.28	0.033	98	4
6.25	0.684	6.18	0.028	99	5
3.12	0.411	3.22	0.013	103	4
1.56	0.254	1.62	0.01	104	6
0.78	0.176	0.85	0.005	108	6
0.391	0.13	0.39	0.006	99	16
0.195	0.112	0.21	0.007	106	36
0.098	0.113	0.21	0.009	211	46
0	0.089	NA	NA	NA	NA

The lowest limit of quantification (LLOQ) of urine soluble cMet was experimentally determined to be 0.78 ng/ml along the calibration curve. This value showed the acceptable level of accuracy and precision in both batches of the ELISA kit.

Supplementary Table S3. Intra-assay and inter-assay Accuracy and Precision of LLOQ

Concentration (ng/ml)	Operator	Intra-assay (n=6)		Inter-assay (n=18)	
		Accuracy (%)	Precision (CV%)	Accuracy (%)	Precision (CV%)
0.78	1	100	8	101	9
	2	108	6	105	5

To confirm 0.78 ng/ml as the LLOQ, intra-assay (within an assay) and inter-assay (between assays) assessment were conducted by two operators, as indicated by the table above. The tested concentration was marked with the acceptable accuracy and coefficient of variation for precision.

Supplementary Table S4. Dilution Linearity of Human Urine Samples

Sample	Dilution	Measured (ng/ml)	Expected (ng/ml)	% Expected	R^2
BRMKID431	1	41.304	41.304	100	0.99
	1/3	15.932	13.768	116	
	1/9	5.851	4.589	127	
	1/27	1.829	1.530	120	
BRMKID487	1	33.22	33.220	100	0.99
	1/3	10.455	11.073	94	
	1/9	3.332	3.691	90	
	1/27	1.028	1.230	84	
BRMKID409	1	5.816	5.816	100	0.99
	1/3	2.093	1.939	108	
	1/9	0.511	0.646	79	
	1/27	0.077	0.215	36	
BRMKID381	1	3.411	3.411	100	0.99
	1/3	1.268	1.137	112	
	1/9	0.346	0.379	91	
	1/27	0.043	0.126	34	
BRMKID398	1	2.274	2.274	100	0.99
	1/3	0.873	0.758	115	
	1/9	0.186	0.253	74	
	1/27	NA	0.084	NA	
BRMKID567	1	2.185	2.185	100	0.99
	1/3	0.75	0.728	103	
	1/9	0.204	0.243	84	
	1/27	NA	0.081	NA	

Supplementary Figure S1. Parallelism of Human Urine Samples

Six human urine samples were randomly obtained from patients and evaluated for parallelism. For parallelism, the optical density of the two samples (BRMKID 481 and BRMKID 487) and their diluted concentrations was plotted against the soluble cMet calibration curve. Parallelism is indicated by the figure above, suggesting that the natural cMet in the urine samples behaves similarly to the calibration curve. Taken together, the results demonstrate that there is no significant matrix effect observed in the human urine samples.

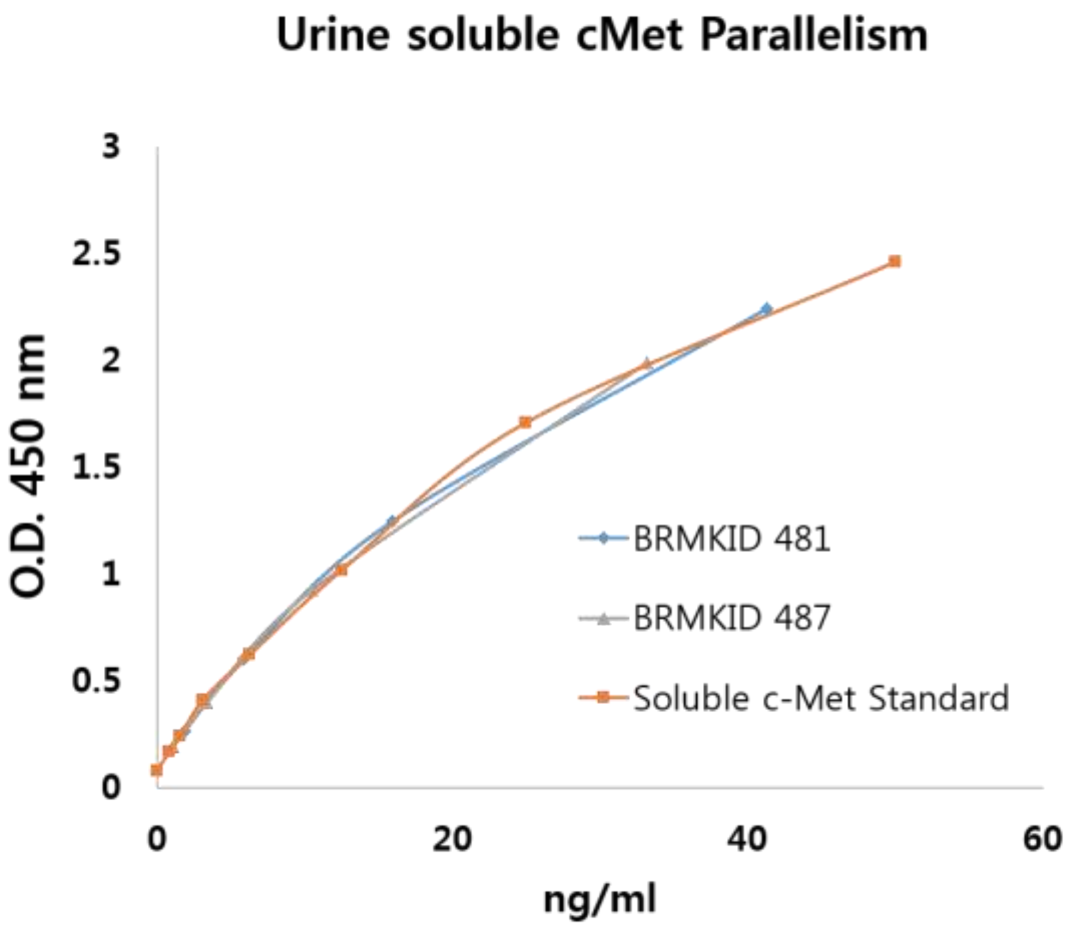

Supplementary Figure S2

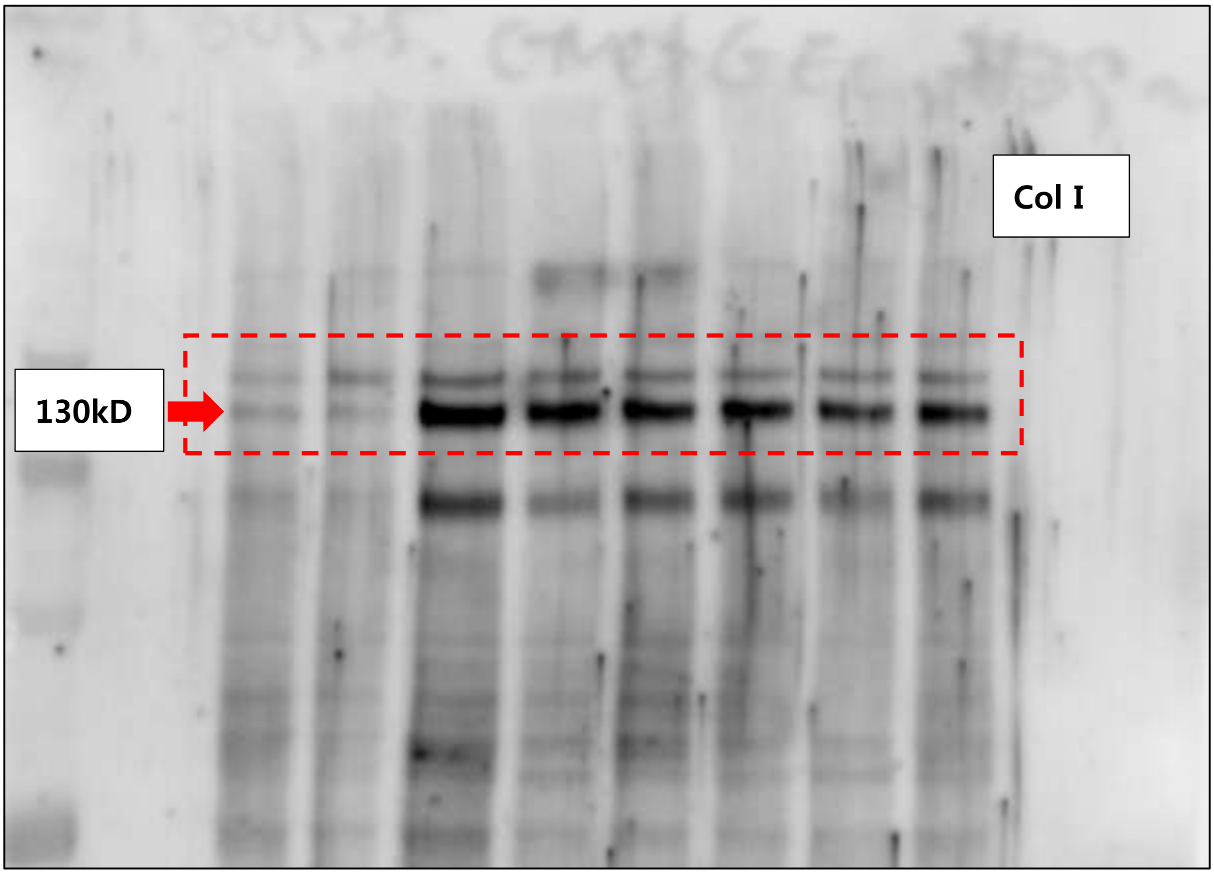

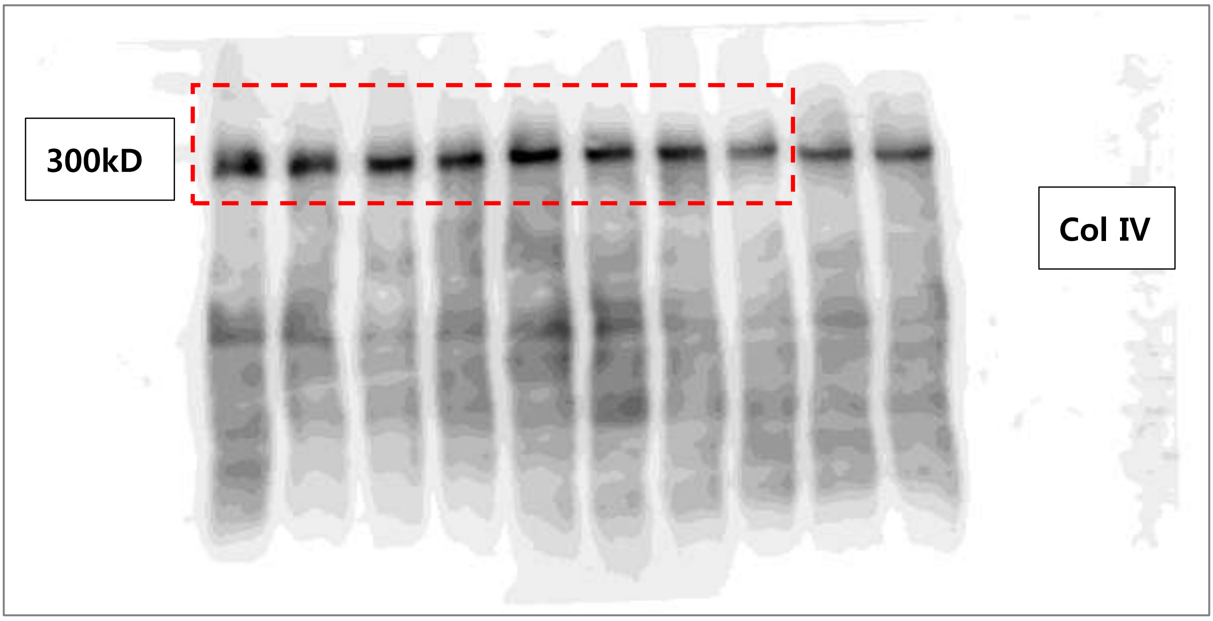

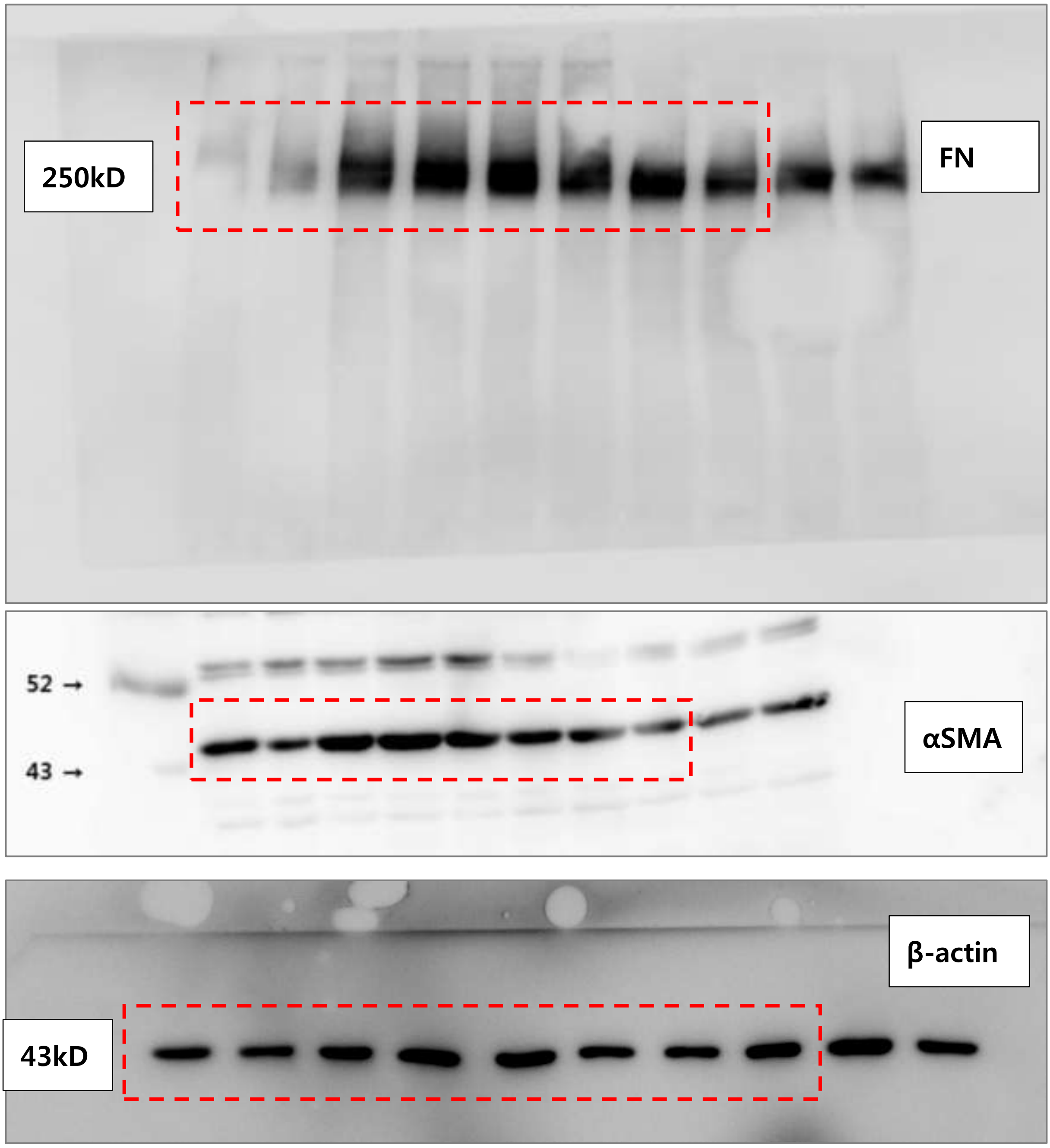

Supplementary Figure S2. Full length blots of Figure 5A. Red dotted lines show the cropping locations.

Supplementary Figure S3

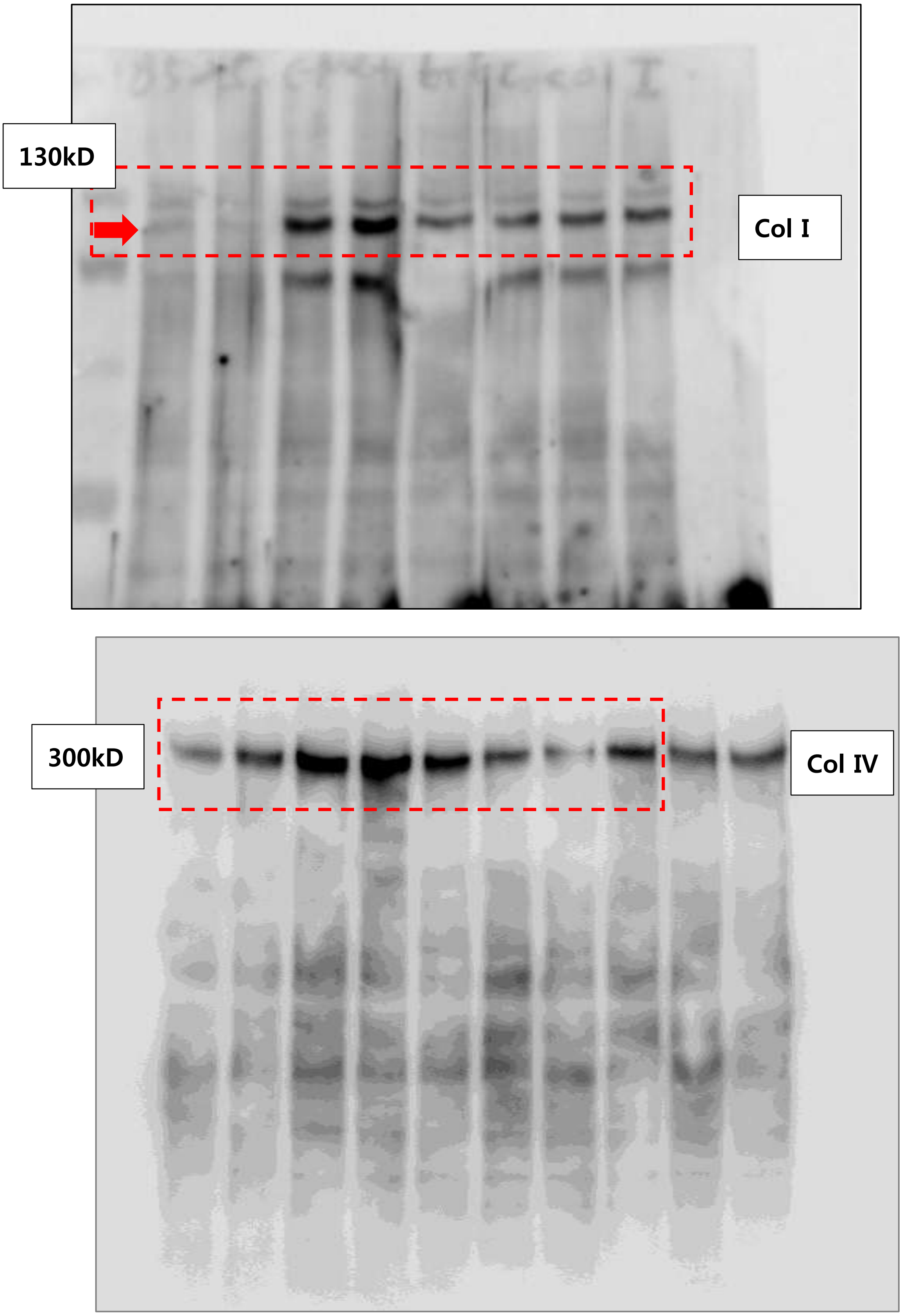

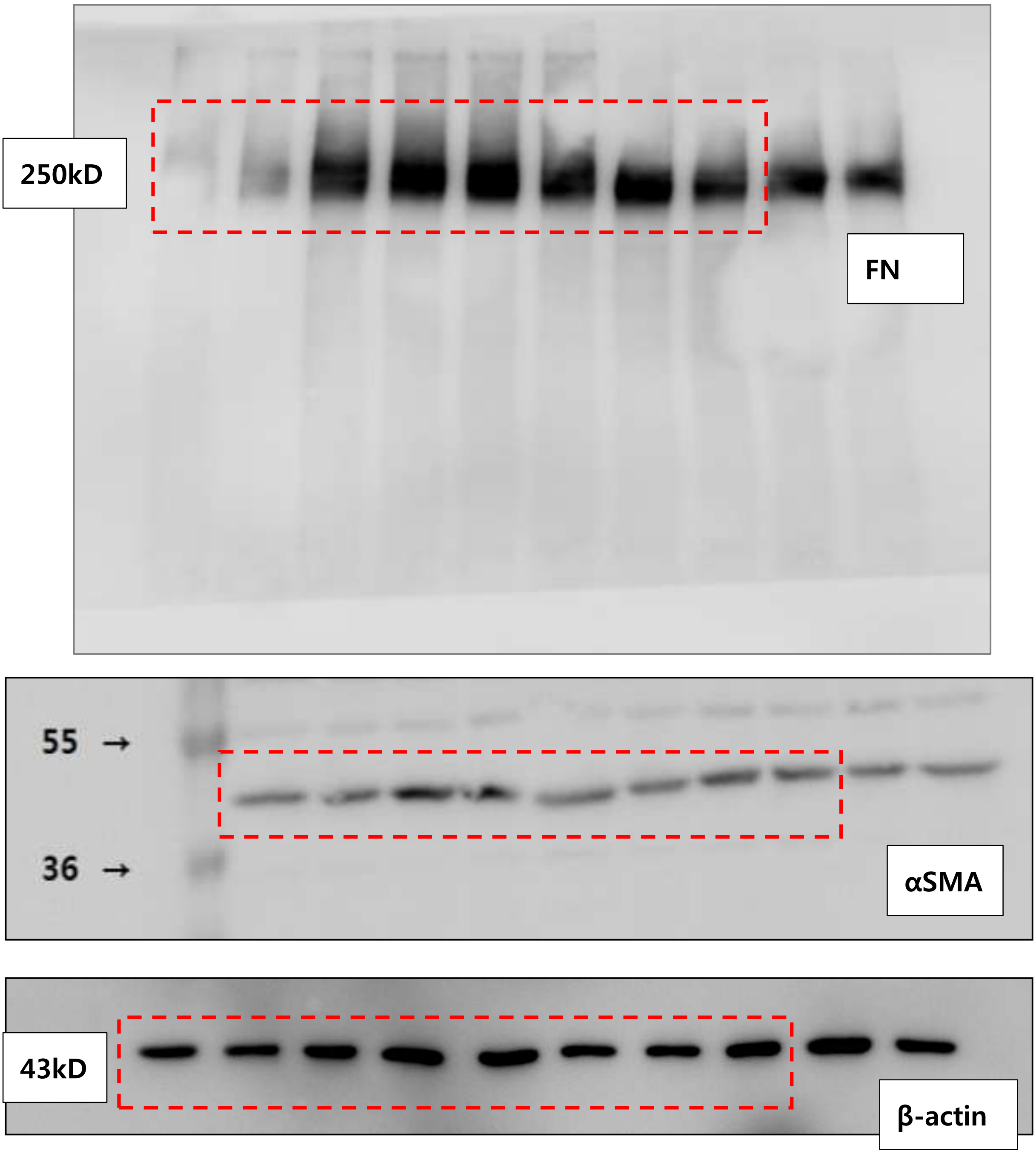

Supplementary Figure S3. Full length blots of Figure 5B. Red dotted lines show the cropping locations.
